# N. Y. Society for the Relief of Widows and Orphans of Medical Men

**Published:** 1855-02

**Authors:** 


*N. Y. Society for the Relief of Widows and Orphans of Medical Men.* —This beneficial association recently held its annual dinner at the Astor House. From the statement of Dr. Wood, the President, we learn that "instituted in 1842, its capital is now $17,000, securely invested in bonds and mortgages, at seven per cent. on improved property in New York and Brooklyn; the ground in every instance being worth the amount loaned. Receipts from all sources during the year (including interest), $1797 15; expenses, $318 25. One family is supported by the society." The receipts on the occasion of the dinner were large, amounting to $1250. The dinner expenses ($630) were paid by private subscription. H.

